# Applicability of Different Electronic Record Types for Use in Patient Recruitment Support Systems: Comparative Analysis

**DOI:** 10.2196/13790

**Published:** 2021-09-21

**Authors:** Björn Schreiweis, Antje Brandner, Björn Bergh

**Affiliations:** 1 Institute for Medical Informatics and Statistics Kiel University and University Hospital Schleswig-Holstein Kiel Germany; 2 Center for Information Technology and Medical Engineering University Hospital Heidelberg Heidelberg Germany

**Keywords:** clinical trials, patient recruitment support system, PRSS, electronic medical record, EMR, electronic health record, EHR, personal health record, PHR, personal enterprise health record, PEHR, clinical trial recruitment support system, CTRSS.

## Abstract

**Background:**

Clinical trials constitute an important pillar in medical research. It is beneficial to support recruitment for clinical trials using software tools, so-called patient recruitment support systems; however, such information technology systems have not been frequently used to date. Because medical information systems' underlying data collection methods strongly influence the benefits of implementing patient recruitment support systems, we investigated patient recruitment support system requirements and corresponding electronic record types such as electronic medical record, electronic health record, electronic medical case record, personal health record, and personal cross-enterprise health record.

**Objective:**

The aim of this study was to (1) define requirements for successful patient recruitment support system deployment and (2) differentiate and compare patient recruitment support system–relevant properties of different electronic record types.

**Methods:**

In a previous study, we gathered requirements for patient recruitment support systems from literature and unstructured interviews with stakeholders (15 patients, 3 physicians, 5 data privacy experts, 4 researchers, and 5 staff members of hospital administration). For this investigation, the requirements were amended and categorized based on input from scientific sessions. Based on literature with a focus on patient recruitment support system–relevant properties, different electronic record types (electronic medical record, electronic health record, electronic medical case record, personal health record and personal cross-enterprise health record) were described in detail. We also evaluated which patient recruitment support system requirements can be achieved for each electronic record type.

**Results:**

Patient recruitment support system requirements (n=16) were grouped into 4 categories (consent management, patient recruitment management, trial management, and general requirements). All 16 requirements could be partially met by at least 1 type of electronic record. Only 1 requirement was fully met by all 5 types. According to our analysis, personal cross-enterprise health records fulfill most requirements for patient recruitment support systems. They demonstrate advantages especially in 2 domains (1) supporting patient empowerment and (2) granting access to the complete medical history of patients.

**Conclusions:**

In combination with patient recruitment support systems, personal cross-enterprise health records prove superior to other electronic record types, and therefore, this integration approach should be further investigated.

## Introduction

Clinical trials constitute an important pillar in medical research. They strongly rely on efficient and sufficient patient cohort recruitment. However, it often proves difficult to (1) complete recruitment in time, (2) achieve the desired number of recruits, and (3) remain within budget [1,2], which altogether jeopardizes the overall success of trials.

The use of patient recruitment support systems has been considered as a measure to overcome these issues. Patient recruitment support systems are information technology apps that are connected to existing care or research information technology systems to automatically or semiautomatically scan for potential trial candidates based on predefined inclusion and exclusion criteria. Patient recruitment support system could considerably improve the number of patients recruited and the time required for the recruitment process [3-5]. Additionally, Köpcke et al [5] reported that patient recruitment support systems can prevent studies from enrolling noneligible candidates. Although various benefits of information technology–based patient recruitment support systems have been identified, a recent study [6] found that information technology support still plays a minor role in the process of screening patients for recruitment support. Still, the integration of data from patient care with patient recruitment support systems is an important requirement described in several studies [7-15].

The underlying data collection method of a given medical information system strongly influences the gains that can be obtained from the use of a patient recruitment support system. Patient recruitment support systems have initially been integrated with either care electronic medical records (EMR) operated within a single institution—often with the hospital’s EMR and a platform specially designed for research [7-19].

Shared care records have been implemented in various projects over the past decades [20]. The evolution of these types of patient records created new possibilities to improve patient recruitment based on a holistic patient history. The integration of data from several health care institutions increases the amount of available medical data for a specific patient. Furthermore, along with the introduction of shared care records, the possibility for patient participation and empowerment evolves, which again can substantially improve recruitment rates into clinical trials by either providing additional patient-centric information or by shared decision-making.

A detailed comparison of gains achieved by different patient recruitment support systems in conjunction with different types of electronic medical or patient records has not yet been published. The aim of this work was, hence, to (1) define requirements for successful patient recruitment support system deployment and (2) differentiate and compare patient recruitment support system–relevant properties of different electronic record types for the purpose facilitating precise descriptions of the benefits that can be achieved and to provide hints for successful implementation projects.

## Methods

### Requirements for Patient Recruitment Support Systems

In a prior investigation [21], we gathered requirements for patient recruitment support systems through a literature analysis and unstructured interviews with 15 patients, 3 physicians, 5 data privacy experts, 4 researchers, and 5 hospital administration staff members. We identified 13 requirements (Table 1).

In this study, the original 13 requirements were amended with 3 additional requirements based on expert feedback from scientific sessions. A total of 16 requirements were grouped into categories and compared by 2 of the authors in a discussion-based consensus process.

**Table 1:** List of requirements [21] that constituted the basis for this study.

Requirement	Description
Patient allows for contact with PI^a^	Patients can choose whether the PI is allowed to contact the patient about the possible participation in a certain trial
Manage informed consent	Patients can manage their own informed consent somehow, eg, by using a web portal
Information whether informed consent available or not	The information whether patient informed consent can be retrieved from the record type
“Physician cannot see if I fit or not”	Patient consent is required for the physician to be notified about possibly eligible patients.
List of all trials for which a patient is potentially eligible	A list of all trials for which a patient is possibly eligible for participation can be displayed
See all patients that fit “my trial”	A list of patients who are possibly eligible for participation in a specific trial can be displayed
Get notified when new patient matching “my trial” is found	A notification can be sent to the PI when a new possibly eligible patient is found for a specific trial
Documentation of trial inclusions	The documentation of patient trial recruitment status is possible
Matching patient-level data with eligibility criteria	An algorithm can be executed to match the trial protocols’ inclusion and exclusion criteria with patient-level data in order to find possibly eligible patients.
Implement trial protocol	The electronic, machine-readable representation of a trial protocol can be generated.
See all trials in institution	A list of trials performed within a health care institution can be displayed.
No extra documentation required	All data previously recorded in any health care provider organization’s EMR^b^ are fully integrated and thus available without requiring (additional) re-documentation.
Data integration with EMR or EHR^c^	Data entered in an EMR or EHR are integrated with the analyzed patient record type.

^a^PI: principal investigator.

^b^EMR: electronic medical record.

^c^EHR: electronic health record.

### Types of Patient Records

#### Overview

As patient-centered health care involves collaborative treatment by more physicians and physician networks, new types of patient and health records have been developed and implemented. At first, hospitals and general practitioners implemented EMRs [22]. Because of increasing needs to exchange health care information, electronic health records (EHRs) emerged [3]. In Germany, a special EHR that contain only health information from a single medical case called *Elektronische Fallakte* (electronic medical case record, EMCR) [23,24] was defined.

Patient empowerment—having patients in a central position regarding their treatment, leading to the idea of patients being managers of their own health—is important. The World Health Organization defines patient empowerment as “a process by which people, organizations and communities gain mastery over their affairs [25].” Thus, the development of records that patients can use to manage their own health care information resulted in the development of personal health records (PHRs) [22]. Patient empowerment or patients as health managers in combination with data integration in EHRs then resulted in the personal cross-enterprise health record (PEHR) [26]. Patients can manage health care data that they either provide themselves or that are provided by their health care providers.

These 5 types of electronic patient or medical records are differentiated in this work. Differences exist regarding (1) data sovereignty, (2) number of involved health care provider institutions, (3) time frame of data storage, (4) the intended use scenario, (5) whether the records are physician- or patient-moderated, (6) whether professional portals are used, (7) whether patient portals are part of the record type, (8) whether the system has a module for seeking consent, and (9) how data are integrated into the record (Table 2).

**Table 2:** Record types and their specific attributes.

Attribute	EMR^a^	EHR^b^	EMCR^c^	PHR^d^	PEHR^e^
Data sovereignty	Health care professionals (ie, physicians)	Health care professionals (ie, physicians)	Health care professionals (ie, physicians)	Patients/citizens	Patients/citizens
Number of health care provider institutions involved	One single institution	Multiple (cross-institutional)	Multiple (cross-institutional)	N/A^f^	Multiple (cross-institutional)
Time frame	Longitudinal (life-long)	Longitudinal (life-long)	Temporary (distinct medical episode and time frame)	Longitudinal (life-long)	Longitudinal (life-long)
Intended use scenario	Clinical/administrative information and documentation	Health care information exchange in-between provider organizations	Health care information exchange in-between provider organizations	Patients’ online-repository for all health care related information in one place including patients’ self-documentation and copied information	Health care information between providers and patients; integration of sensors and monitoring (Home Care); patient empowerment; patient self-documentation; patient reported outcome and experience measures
Moderation	Physician moderated	Physician moderated	Physician moderated	Patient moderated	Patient moderated
Professional portal	N/A	Possible (not required)	Possible (not required)	N/A	Required
Patient portal	N/A	N/A	N/A	Required	Required
Module for consent creation	Within primary system	Within primary systems	Within primary systems	Mostly not available, otherwise token-based	Patient portal
Data integration into record	N/A	Automatic and manual	Automatic and manual	Mostly manual	Automatic and manual

^a^EMR: electronic medical record.

^b^EHR: electronic health record.

^c^EMCR: electronic medical case record.

^d^PEHR: personal health record.

^e^PEHR: personal cross-enterprise health record.

^f^N/A: not applicable.

#### EMR

**Figure 1:**
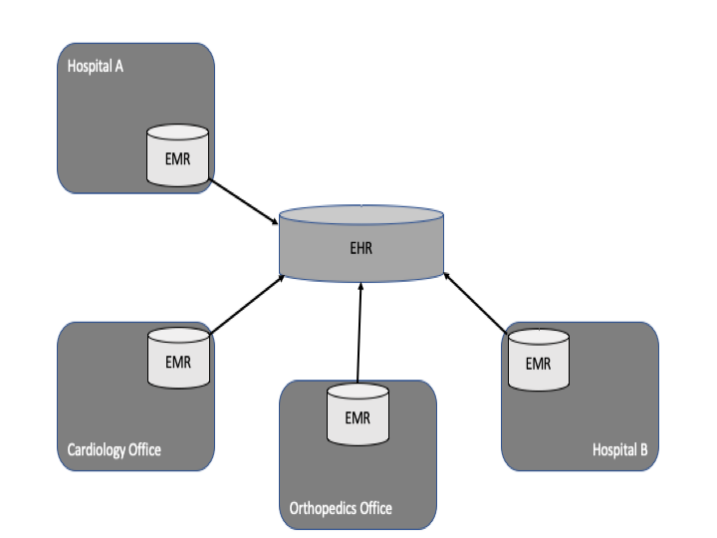
Integration of electronic medical records (EMRs) in an electronic health record (EHR) that includes data from several institutions.

EMR is the typical (electronic) record within a single health care institution (Figure 1). An EMR is solely based on information documented within the health care institution (duty of medical documentation and for administrative purposes) and information brought by the patient. That includes, but is not limited to, patient demographic information, diagnoses, therapies, medications, laboratory results, and various types of images (eg, magnetic resonance imaging and computed tomography images). Thus, the EMR is part of the hospital information system. Information and documents brought by the patient are scanned (as PDF, TIFF, or similar formats) or imported, in the case of electronic data [3], to the institution’s patient record archive. Data are often documented in semi- or unstructured forms or text documents [27], but structured values, such as lab results, may be available. As the EMR is a physician-moderated record, data sovereignty remains with health care professionals.

#### EHR

An EHR is an automatic and manual integration of several EMRs to a single record that link health care information from multiple health care institutions (Figure 1). Physicians both manage and moderate the content. EHRs can be used for different use cases or purposes such as sharing data in a health care network, building a regional health record, or sharing data for research purposes (eg, [17]). In all of these scenarios, physicians moderate the content, and in doing so, maintain data sovereignty. In an EHR, access to patient data is granted based on integrated treatment contracts between institutions, which are managed within an institution’s primary systems. Access to physicians can be provided from primary systems or via professional portals. EHRs are longitudinal records that integrate medical data from—ideally—all health care institutions at which the patient has been treated. Implementations often focus on health care regions or integrated care networks [28,29]. Data are integrated to support patient treatment; Thus, data exchange is limited to PDFs and other unstructured documents with narratives. Literature on the amount of structured data in EHRs was not available at the time of this study. In the United States, the *meaningful use campaign* propagates to use documents based on HL7 Clinical Document Architecture, which are semistructured documents describing patient history [30].

#### EMCR

**Figure 2:**
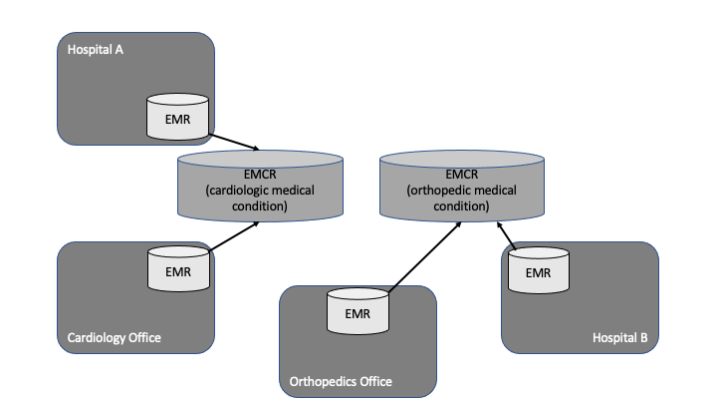
Integration of electronic medical records (EMRs) in electronic medical case record (EMCR) depending on the medical case (either cardiology or orthopedic).

As a special type of EHR, an EMCR represents a record for a distinct medical condition (eg, cardiac stroke, chronic diseases such as diabetes, etc), and data can be duplicated and shared with every health care provider involved in the patient’s treatment [24]. Another implementation uses a central repository, which allows health care providers involved in the treatment of the condition to access and edit content. In both implementations, data integration can be manual or automatic. Physicians decide which consent is regarded relevant for the record (physician moderated), and data sovereignty remains with them. However, patient consent is managed within each institution’s primary system separately. Records are closed after completion of treatment; therefore, the record is only temporary. Access across several medical conditions is not possible, regardless whether data are centralized or decentralized (Figure 2). Usually, physicians access records in their primary systems or via professional portals.

#### PHR

**Figure 3:**
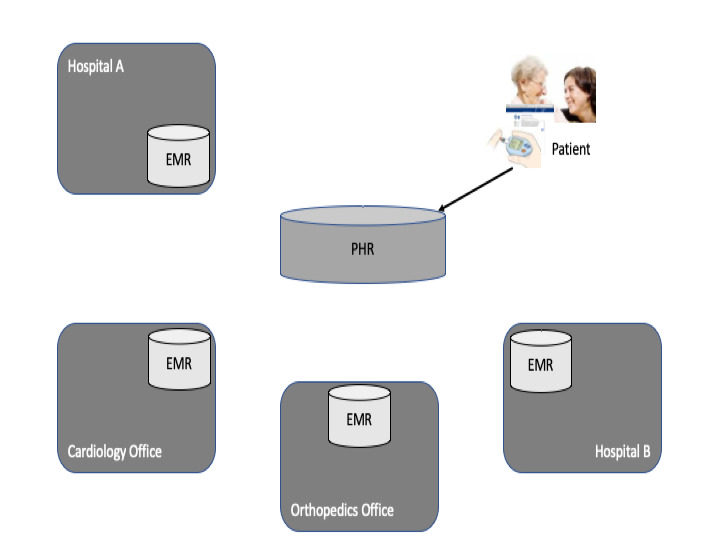
Integration of electronic medical records (EMRs) and personal health record (PHRs), as found in most PHRs.

The PHR is similar to the EHR except that patients set up, access, and manage the record themselves (patient-moderated) instead of this being done by the physicians involved in their care [22]. Accordingly, patients maintain data sovereignty. PHRs often lack integration with patients’ EMRs and EHRs (Figure 3); therefore, patients have to manually enter or upload all data they want to include in their PHR. The intended use scenario of PHRs is to provide patients with a web-based repository for managing their life-long health care–related information in a single place, including self-documented and copied information; therefore, patient portals or mobile apps are provided as user interfaces for patients. Consenting to give access to health care providers is seldom possible and, if available, based on access tokens for providers.

#### PEHR

A PEHR is a combination of EHR and PHR that allows for patients to permit access to or storage of the information in their PEHR by health care providers involved in their care (patient-moderated record) [26]. The idea is to empower patients by providing a means for them to access their medical data and to decide who can read or write medical information. Thus, data sovereignty remains with the patient. As it is a longitudinal record, data (including home care, monitoring devices, self-documentation as well as patient reported outcome and experience measures) are integrated manually or automatically during the entirety of patients’ lives. Patients use a patient portal or mobile app to access their health information, to manage the access policies for health care providers, and to consent to secondary use. Physicians access data using a professional portal, which can be fully integrated into physicians’ EMR systems. The professional portal ensures that data can be visualized directly and prevents the duplication of data because information is not downloaded. Thus, it is easier to enforce data deletion and withdrawals of access to the patient’s health information [26].

**Figure 4:**
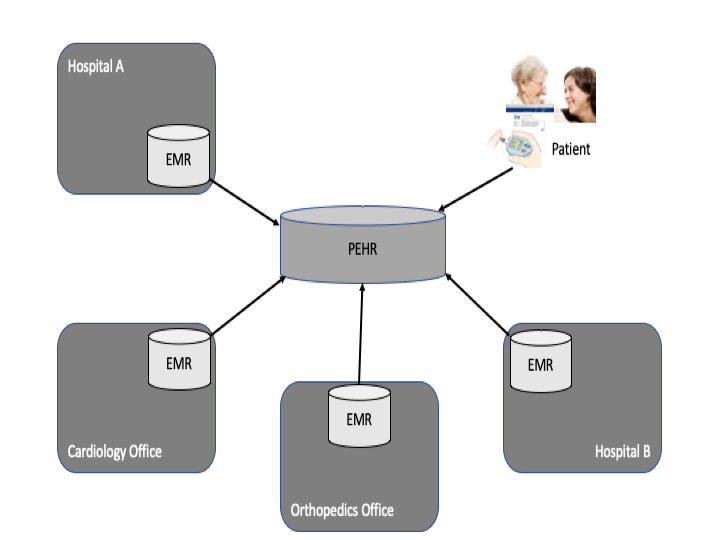
Integration of electronic medical records (EMRs) with a personal cross-enterprise health record (PEHR).

The PEHR is integrated with multiple health care providers’ EMRs using international interoperability standards. Data in the record can be structured, semistructured, or unstructured and are exchanged between EMRs and PEHRs in containers called documents. Health Information Exchange is implemented using profiles from the initiative Integrating the Healthcare Enterprise [26,31] (Figure 4).

### Combination of Patient Recruitment Support System Requirements and Types of Patient Records

We described which patient recruitment support system requirements can be achieved for each electronic record type based on the architectural design. Information about the implementation of requirements was referenced from literature wherever possible.

## Results

### Amendment and Categorization of Patient Recruitment Support System Requirements

The list of requirements identified in [21] was amended with addition of 3 requirements (Table 3, Multimedia Appendix 1): (1) obtaining patient consent in a timely manner (Obtain consent on short notice), (2) requiring informed patient consent for the use of health data in the patient recruitment support system (Informed consent required to use health data in patient recruitment support systems); and (3) completeness of the group of patients eligible and represented in the record type (Completeness concerning eligible patients). The requirements were grouped into 4 categories: consent management (CM), patient recruitment management (PRM), trial management (TM), and general requirements (GR). Consent management addresses all requirements with respect to patient consent. Requirements grouped in patient recruitment management include those with direct impact on patient recruitment. TM includes requirements about trials in general and prerequisites for patient recruitment. GR describes generic requirements.

**Table 3:** Amended list of requirements.

Category and requirement^a^			Description
**CM^b^**			
	CM1	Patient allows for contact with PI^c^	Patients can choose whether the PI is allowed to contact the patient about the possible participation in a certain trial.
	CM2	Manage informed consent	Patients can manage their own informed consent somehow, eg, by using a web portal.
	CM3	Information whether informed consent available or not	The information whether the patient informed consent can be retrieved from the record type.
	CM4	“Physician cannot see if I fit or not”	Patient consent is required for the physician to be notified about possibly eligible patients.
	CM5	Obtain consent on short notice^a^	Patients can easily be contacted and document their consent in a way that it is machine interpretable
	CM6	Informed consent required to use health data in patient recruitment support systems^a^	The patient recruitment support system can only access health data of patients that previously gave their informed consent
**PRM^d^**			
	PRM1	List of all trials for which a patient is potentially eligible	A list of all trials a patient is possibly eligible for participation can be displayed.
	PRM2	See all patients that fit “my trial”	A list of patients who are possibly eligible for participation in a specific trial can be displayed.
	PRM3	Get notified when new patient matching “my trial” is found	A notification can be sent to the PI when a new possibly eligible patient is found for a specific trial.
	PRM4	Documentation of trial inclusions	The documentation of patient trial recruitment status is possible.
	PRM5	Matching patient-level data with eligibility criteria	An algorithm can be executed to match the trial protocols’ inclusion and exclusion criteria with patient-level data in order to find possibly eligible patients.
**TM^e^**			
	TM1	Implement trial protocol	The electronic, machine-readable representation of a trial protocol can be generated.
	TM2	See all trials in institution	A list of trials performed within a health care institution can be displayed.
**GR^f^**			
	GR1	No extra documentation required	All data previously recorded in any health care provider organization’s EMR^g^ are fully integrated and thus available without requiring (additional) redocumentation.
	GR2	Data integration with EMR or EHR^h^	Data entered in an EMR or an EHR are integrated with the analyzed patient record type.
	GR3	Completeness concerning eligible patients^a^	All patients that are possible eligible from the population are represented in the respective patient record.

^a^Requirements with this superscript were added to the original list of 13 requirements.

^b^CM: consent management.

^c^PI: principal investigator.

^d^PRM: patient recruitment management.

^e^TM: trial management.

^f^GR: general requirements.

^g^EMR: electronic medical record.

^h^EHR: electronic health record.

### Requirements Implemented by Record Type

#### Overview

The requirements that are implementable by the different types of electronic patient records are described record by record followed by a comparative overview (Table 4).

**Table 4:** Requirements for patient recruitment support systems met (✓) by different patient records.

Requirement		EMR^a^	EHR^b^	EMCR ^c^	PHR^d^	PEHR^e^
CM1	Patient allows for contact with principal investigator	—^f^	—	—	—	✓
CM2	Manage informed consent	—	—	—	✓	✓
CM3	Information whether informed consent available or not	✓	—	—	—	✓
CM4	“Physician cannot see if I fit or not”	—	—	—	—	✓
CM5	Obtain consent on short notice	✓^g^	✓^g^	✓^g^	✓	✓
CM6	Informed consent required for health data use in patient recruitment support system	—	—	—	—	✓
PRM1	List of all trials for which a patient is potentially eligible	—	—	—	✓	✓
PRM2	See all patients that fit “my trial”	✓	✓	✓	—	✓^g^
PRM3	Get notified when new patient matching “my trial” is found	✓	✓	✓	—	✓^g^
PRM4	Documentation of trial inclusions	✓	✓	✓	—	✓
PRM5	Matching patient-level data with eligibility criteria	✓	✓	✓^g^	✓	✓
TM1	Implement trial protocol	✓	✓	✓	✓	✓
TM2	See all trials in institution	✓	✓	✓	—	✓
GR1	No extra documentation required	—	✓^g^	—	—	✓
GR2	Data integration with an electronic medical or health record	✓	✓	—	—	✓
GR3	Completeness concerning eligible patients	—	✓	—	✓^g^	✓

^a^EMR: electronic medical record.

^b^EHR: electronic health record.

^c^EMCR: electronic medical case record.

^d^PHR: personal health record.

^e^PEHR: personal cross-enterprise health record.

^f^Requirement not met.

^g^Requirement only partially met.

#### EMR

EMRs can implement clinical trial protocol (TM1) either with integrated functionality or by extending functionality [16,18,19,32-35]. Because the purpose of EMRs is to facilitate documentation of patients’ medical histories for duty of medical documentation and billing purposes, the integration of patients’ health care information (GR2) is achieved by implementing a patient recruitment support system with an EMR. By providing the medical history of patients who are treated by the health care provider in the EMR, the system allows medical history information to be searched and matched with clinical trials eligibility criteria (PRM5). Within the EMR, additional information, such as patient consent, can be stored and archived. Thus, the information about whether patients have consented to the usage of their clinical information for research purposes (broad consent) or for a certain research project or clinical trial (informed consent) can be obtained through EMRs (CM3). In the event that named informed consent is not yet available, it can only be obtained from the patient as long as the patient is with the institution. Therefore, obtaining patient consent on short notice is only possible in some cases (CM5). If worklists are possible in the EMR system, patients that fulfill eligibility criteria can be shown on a list (PRM2), and the treating physician can be informed about new hits (eg, [33]) (PRM3). In university hospitals or other institutions involved in research, an extension that allows patient inclusion into clinical trials to be documented is available (PRM4) (eg, [34,35]). The implementation of a trial portal for managing trials can help institutions monitor all trials performed (TM2) [11]. Management of the trials also allows for the definition of eligibility criteria (TM1) [11]. EMR patient recruitment support system integration meets 8 out of 16 (50%) requirements fully and 1 requirement partially.

#### EHR

For EHRs, functionality for discovering and listing all patients that fulfill eligibility criteria for a certain physician’s or principal investigator’s specific clinical trial can be implemented, as EHRs are physician-controlled patient records (eg, [17]) (PRM2). The same applies to matching new patients to a specific clinical trial (PRM3). For documentation of trial inclusions, EHRs require the same additional module as EMRs: a screening module [34,35] (PRM4); however, this can be implemented. The implementation of a trial protocol is also possible in EHRs (TM1) and necessary to be able to execute patient–eligibility criteria matching (PRM5). Extra documentation, to support patient eligibility checks, is not required (GR1), as all relevant patient medical information is already integrated in the EHR (GR2). Thus, GR1 is only partially met. The integration of patient recruitment support systems with EHRs includes data from at least 1 EMR but can also include data integrated from several EMRs to the EHR. Therefore, it can be assumed that eligible patients are within the EHR and completeness is achievable (GR3). Complementary use of the module for trial protocol management with user authorization services also allows for the identification and visualization of trials per institution (TM2). Obtaining patients’ informed consent (CM5) for trial participation can be difficult, if the patient is not affiliated with any health care institution participating in the EHR during the recruitment phase. Thus, CM5 is only partly met. Thus, 8 out of 16 (50%) requirements can be completely met, and 1 requirement can be partially met by integrating a patient recruitment support system with an EHR.

#### EMCR

In an EMCR the integration with a patient recruitment support system allows physicians and principal investigators to see all patients that fit a particular clinical trial (PRM2) and be notified when a new patient match for one of the physician’s or principal investigator’s clinical trials is found (PRM3). If patients are included in a clinical trial, the documentation of trial inclusions can be integrated with the EMCR (PRM4). The inclusion status will only be available for trials for the same medical condition because each medical condition is documented in a distinct EMCR. This also applies to data required for matching patients with a certain trial. Therefore, the execution of patient–eligibility criteria matching is only partially possible (PRM5). The implementation of trial protocols is possible (TM1). The inclusion of physicians or health care institutions and trial protocol management also allow for an overview of trials performed within an institution (TM2). Patients’ informed consent can be obtained on short notice (CM5), since a match can be found whether a patient is treated in a participating institution or not. Therefore, CM5 is only partly met. The integration of patient recruitment support systems with EMCRs meets 5 out of 16 (31%) requirements fully and 2 requirements partially.

#### PHR

Patient recruitment support system integration with PHRs allows patients to have an overview of the clinical trials for which they are possibly eligible (PRM1) and in which they participate. Patients can manage their own informed consent (CM2) about (1) being contacted regarding a certain clinical trial and (2) participating in a specific clinical trial (CM5). As a PHR usually is not integrated with EMR or EHR systems, the physician cannot see if a patient is possibly eligible for their trial (PRM2). The physician or principal investigator will only get notified after a patient consents to receiving more information or being contacted. Implementation of trial protocols (TM1) using an PHR is possible [36-39]. As these PHRs are implemented for patient-eligibility matching, the execution of patient–eligibility criteria matching (PRM5) is possible [36-39]. As it is most likely that not all possibly eligible patients use the PHR, it might be difficult to achieve completeness concerning eligible patients (GR3). Five out of 16 (31%) requirements for patient recruitment support systems can be completely met by integrating PHR and patient recruitment support system, and 1 requirement (GR3) is only partly met.

#### PEHR

A patient recruitment support system implementation integrated with a PEHR gives patients an overview of all clinical trials for which they are possibly eligible (PRM1). For each clinical trial, the patient can then decide whether the principal investigator of the trial is allowed to contact them about trial inclusion (CM1). This functionality implicitly mentions the management of patients’ informed consent (CM2). Consent management in a PEHR allows the information to be retrieved whether informed consent has been obtained or not (CM3) by retrieving patients’ policies and enforcing them (ie, allowing or denying a certain transaction) [40]. But it is also possible for patients who do not want their data to be available to the patient recruitment support system to prohibit data use, since the patient recruitment support system requires that patients consent to the use of their data (CM6). From a patient perspective, the decision whether a physician or principal investigator is informed about eligibility status is also based on the patient’s consent; therefore, a physician cannot automatically see if the patient fits the trial or not (CM4). Thus, the physician or principal investigator can see all patients who consented to being contacted and fit the trial but not those who did not consent, which results in physicians possibly only seeing a portion of eligible patients (PRM2). A notification for new patients who are possibly eligible for the trial is also possible only if patients consented (PRM3). Patients’ informed consent can be obtained at all times because patients do not have to be with the institution but can give their consent via the PEHR (eg, via patient portal or mobile app) (CM5). The inclusion of a patient can be documented in the PEHR (PRM4), and afterward, can be used as data for eligibility screening in future clinical trials. To find trials that match a patient or patients who match trials, the implementation of the trial protocol (TM1) is necessary. This functionality is possible by integrating the PEHRs with patient recruitment support systems. Thus, patient–eligibility criteria matching (PRM5) is also a given functionality. Because the PEHR integrates data from EMRs (GR2) and a patient’s self-documented data, neither the patient nor the physician has to perform extra documentation (GR1) to match a patient with clinical trial eligibility criteria. If all clinical trials for a given institution are implemented in the patient recruitment support system of a PEHR, an overview of all clinical trials performed at the institution (TM2) is possible. The PEHR, as a regional record, provides completeness concerning eligible patients as data from of all individuals in the region are contained within the PEHR (GR3). Thus, patient recruitment support system–PEHR integration allows for 14 out of 16 (88%) requirements to be fully met and for 2 (PRM2, PRM3) additional requirements to be met, albeit with restrictions.

## Discussion

### Principal Results

Our evaluation identified that only 1 requirement can be fully implemented in all 5 types of electronic patient records—the requirement to have functionality for the implementation of trial protocols (TM1). All other requirements could be implemented in 1 to 4 records.

Only PEHR–patient recruitment support system integration allowed for all requirements to be at least partially met (14/16, 88%), followed by EMR–patient recruitment support system integration (8/16, 50%) and EHR–patient recruitment support system integration (8/16, 50%). The integrations with the least requirements being met were PHRs (5/16, 31%) and EMCRs (5/16, 31%). Possible explanations for these results follow.

An EMR is limited to health care information documented during treatment within the institution and information brought by the patient. Integration with other health care providers involved in the patient’s treatment cycle is missing. Thus, a holistic view of the patient’s health care information is almost impossible unless the patient is treated only at a single institution. In the literature, many examples are given for patient recruitment support systems integrated with EMRs [16,18,19,32-35]. The patient is not able to manage their own information. Consents are persistent within the institution. Both might lead to patient recruitment support system integration with EMRs not meeting patient-centered requirements.

If a patient recruitment support system is integrated with an EHR, an important question is, “who is to be contacted about a patient who matches the eligibility criteria of a trial?” Under German laws, either a member of the patient’s treatment team or patients themselves have to be informed because physicians are bound by medical confidentiality. Consent has to be obtained before the principal investigator or a physician outside the patient’s treatment team can contact a patient and inform them about the trial. Afterward, informed consent for trial inclusion has to be obtained by the principal investigator. One problem is that the patient, at the moment of possible eligibility, might be healthy and not with a physician. The patient would not be able to be included in the trial because they could not be contacted, unless they had consented in advance to be contacted in the case of a trial match. An important benefit of using EHRs over EMRs is the amount of patient data that is available for patient–eligibility criteria matching, as data provided by more than one institution are integrated in an EHR.

With the EMCR, one problem is that medical data are available for a distinct medical case only. Also, the most recent data may only be available with the latest treating health care provider because all former health care providers involved might not be known. The patient’s complete health care information is distributed over multiple EMCRs, with each consisting of information of another distinct medical condition. Clinical trials about more than 1 distinct medical condition or confounding medical conditions might not be possible, because data are documented in different ECRs for the same patient. The next problem for integrating an EMCR with a patient recruitment support system is that the EMCRs are closed after the treatment of the medical condition is finished, either successfully or after the patient has died. After closing the EMCR, the data would no longer be available for a patient recruitment support system but might still be relevant to check for trial eligibility.

When PHRs are integrated with a patient recruitment support system, patients are responsible for entering all information required for matching eligibility criteria, as most PHRs are not integrated with EMRs or EHRs. This can be error-prone, since limited health literacy can result in incorrect documentation. Incorrect data can result in an additional workload for the principal investigator, as data have to be verified [41,42]. If the patient matches a clinical trial and chooses to contact the principal investigator about possible inclusion into the trial, the principal investigator (1) has to enter the patient’s health information again and (2) has to match the patient’s health care information again with the eligibility criteria. Patient recruitment support systems implemented as part of PHRs are often systems that require the patient to enter data every time they want to check whether they fit a clinical trial or not. Persistence of patient medical data depends on the implementation of the system [36-39]. Automatic integration of patients’ health information from EMRs and EHRs with the PHR would lead to PHRs matching almost as many requirements as PEHRs.

The PEHR, as a combination of PHR (patient portal to access health information and manage access to this information [43]) and EHR (professional portal and EMR integration), allows for all requirements to be at least partially fulfilled, when integrated with patient recruitment support systems. Exceptions to fully meeting requirements are PRM2 and PRM3, because they strongly depend on whether patients matching trials consent to being contacted. Thus, these requirements are only partially fulfilled.

With respect to data privacy, there are several options. When patient consent is involved, there are only 2 options: opt-in and opt-out. However, whether opt-in or opt-out is required by data privacy laws does not matter when it comes to patient involvement. The patients can only be involved when they have access to their health information and know where health information is stored and used. Data privacy requirements are part of PRM1 (List of all trials for which a patient is potentially eligible), CM2 (Manage informed consent), CM3 (Information whether informed consent is available or not), and CM4 (“Physician cannot see if I fit or not”). Full access control and control of the use of personal health information by patients themselves necessitates the integration of patient recruitment support systems with either PHRs or PEHRs.

### Limitations

There are many different definitions for EMR, PHR, and especially, EHR available. Thus, we had to pick one for each. Other definitions might lead to different results regarding the requirements met by each record type.

### Comparison With Prior Work

Patient recruitment support systems are, to date, mostly integrated with EMRs [16,18,19,32-35]; however, some patient recruitment support system–PHR integrations exist [36-39]. To the best of our knowledge, no evaluations of patient record types concerning their applicability for patient recruitment support systems had been completed prior to this work.

### Conclusions

Only the integration of a patient recruitment support system with a PEHR environment leads to an implementation with all requirements met. Data integration and use of medical information for research purposes, such as matching eligibility criteria are fully controlled by the individual through consent management. A patient recruitment support system integrated with a PEHR would be a cross-enterprise patient recruitment support system. Further research on patient recruitment support system integration with PEHRs will lead to architectures that allow successful integration.

## Appendix

Multimedia Appendix 1 Table of requirements with examples for each requirement.

## Abbreviations

CM: consent management
EHR: electronic health record
EMCR: electronic medical case record
EMR: electronic medical record
GR: general requirement
PEHR: personal cross-enterprise health record
PHR: personal health record
PRM: patient recruitment management
TM: trial management
